# PET Imaging of a Transgenic Tau Rat Model SHR24 with [^18^F]AV1451

**DOI:** 10.1007/s11307-024-01972-4

**Published:** 2025-01-21

**Authors:** Nisha K. Ramakrishnan, Annie Ziyi Zhao, Stephen Thompson, Selena Milicevic Sephton, David J. Williamson, Tomáš Smolek, Norbert Žilka, Franklin I. Aigbirhio

**Affiliations:** 1 Molecular Imaging Chemistry Laboratory, Wolfson Brain Imaging Centre, Department of Clinical Neurosciences, University of Cambridge, Cambridge, CB2 0QQ UK; 2 Preclinical Imaging Research Laboratory, Anne McLaren Building, 90 Francis Crick Avenue, Trumpington, Cambridge, CB2 0BA UK; 3 Axon Neuroscience R&D Services SE, Dubravska vćesta 9, 811 02 Bratislava, Slovakia; 4 Institute of Neuroimmunology, Slovak Academy of Sciences, Dubravska Cesta 9, 845 10 Bratislava, Slovakia

**Keywords:** Positron emission tomography, Kinetic modelling, Tau rat, Reference tissue model, Autoradiography

## Abstract

**Purpose:**

Positron Emission Tomography (PET) scans with radioligands targeting tau neurofibrillary tangles (NFT) have accelerated our understanding of the role of misfolded tau in neurodegeneration. While intended for human research, applying these radioligands to small animals establishes a vital translational link. Transgenic animal models of dementia, such as the tau rat SHR24, play a crucial role in enhancing our understanding of these disorders. This study aims to evaluate the utility of SHR24 rat model for PET studies.

**Procedures:**

Dynamic PET scans were conducted in male SHR24 rats and their wild-type SHR (SHRwt) littermates using [^18^F]AV1451. Rapid blood sampling and metabolite analysis were performed to acquire input curves. Time activity curves were obtained from various brain regions over 60 min. Blood-based, 2-Tissue Compartment Model (2-TCM) and Logan graphical analysis were used to obtain kinetic modelling parameters. The ability of reference tissue models to predict the binding potential (BP_ND_) were assessed. Autoradiography studies were performed to corroborate the scan data.

**Results:**

Total distribution volume (V_T_) was the best predicted parameter which revealed significantly higher uptake of [^18^F]AV1451 in the cortex (5.8 ± 1.1 vs 4.6 ± 0.7, *P* < 0.05) of SHR24 rats compared to SHRwt rats. Binding potential obtained from 2-TCM was variable, however BP_ND_ from reference tissue models detected significantly higher binding in cortex (0.28 ± 0.07 vs 0.20 ± 0.04, *P* < 0.01 by SRTM) and brainstem (0.14 ± 0.04 vs 0.08 ± 0.02, *P* < 0.01, by SRTM).

**Conclusions:**

With the ability to detect binding of established radioligand [^18^F]AV1451 in these rats, we have demonstrated the utility of this model for assessing aggregated tau neurobiology by PET, with reference tissue models providing potential for longitudinal studies.

**Supplementary Information:**

The online version contains supplementary material available at 10.1007/s11307-024-01972-4.

## Introduction

The development of selective positron emission tomography (PET) radiotracers for tau neurofibrillary tangles (NFTs) represents a significant advancement in neurodegenerative disease research. In Alzheimer’s disease (AD), tau PET imaging outperforms amyloid imaging and other imaging modalities such as MRI in predicting cognitive decline [1]. Many of these breakthrough insights were enabled by the first generation tau radiotracer [^18^F]AV1451, however, it along with [^11^C]PBB3 and THK series radioligands, faced challenges such as off-target binding to MAO A/B or lack of metabolic stability [2–4]. Second generation radioligands such as [^18^F]RO-948, [^18^F]MK-6240, [^18^F]PI-2620, [^18^F]JNJ-311 aim to address these disadvantages, however, off-target binding remains a concern for some [5]. Tau PET imaging research, with the goal of developing radiotracers for the various tauopathies, expressing different protofilament folds, isoforms and species of misfolded tau remains challenging [6] and exciting [7–9].

While the aim is to apply these radioligands for human research studies, a major strength of PET for research is that the same technique and PET radioligands can be applied to both humans and small animals and hence image the same biological targets in animal models as those being studied in humans. This provides a critical translational link from laboratory research in animal models to human disease. Hence transgenic animal models of dementia can be important in furthering the understanding of these disorders, especially given their use in longitudinal and behavioural studies. Despite the excellent affinities of current tau PET radioligands for tau fibrils, their ability to visualize tau NFT in animal models remains limited, with varying success in transgenic mice models where full kinetic modelling is impractical [10, 11]. Notably, PET imaging of tau pathology in a rat model of pure tau pathology is yet to be established, posing a significant limitation for translational dementia research. Rats offer distinct advantages over mice for PET imaging due to i) their larger brains, allowing more accurate quantification of radioactivity concentration in brain regions; ii) feasibility of blood sampling for gold-standard blood-based kinetic analysis, and iii) more favourable for behavioural and cognitive testing.

Of the transgenic rat models expressing human tau [12], most express a mutant form of tau [13, 14] or co-express other misfolding proteins [15, 16]. Three rat models [17–19] from Axon Neuroscience express non-mutated, truncated human tau pathology exclusive to other misfolded proteins. Of these, the SHR24 tau rats [18] selectively express truncated tau consisting of three microtubule-binding domains and a proline rich region (3R tau151-391). These rats develop progressive, age-dependent NFTs in the cortex, satisfying the histopathological criteria used to identify neurofibrillary degeneration in AD, including argyrophilia, Congo red birefringence, and Thioflavin S reactivity. The NFTs start appearing at the age of 9 months, increase with age and shorten the lifespan of these animals to about 16 months. With the presence of NFTs containing human tau, this rat model presents a potential for quantitative, translational PET imaging studies.

This study aimed to utilise the FDA-approved radiotracer for imaging tau [^18^F]AV1451 (also known as [^18^F]T807, [^18^F]Flortaucipir), to assess the utility of SHR24 rat model for assessing aggregated tau neurobiology by PET and for evaluating novel tau PET imaging agents. We performed dynamic PET scans in this rat model and its wildtype with [^18^F]AV1451. The data was evaluated using gold-standard, blood-based kinetic modelling as well as reference tissue models to facilitate future longitudinal studies. In vitro autoradiography along with immuno/fluorescence staining studies were performed to establish binding of the radiotracer to the NFTs.

## Materials and Methods

### Radiochemistry

The radiosynthesis of [^18^F]AV1451 was achieved in two steps following the previously published protocol by the Scott group [20] (see supplementary information).

### Animals

Male SHR24 transgenic tau rats (SHR24) (*n* = 13) and their age-matched wildtype SHR littermates (SHRwt) (*n* = 15) were imported from Axon Neuroscience (Bratislava, Slovak Republic) at 13–14 months old (average weights, SHRwt = 392 g, SHR24 = 261 g) and allowed to acclimatise for a minimum of seven days before the scans. They were housed in Techniplast 2000P IVC cages with play tunnels and aspen bricks as enrichment, on a layer of Aspen bedding at a constant temperature (21 ± 2 °C) and fixed 12 h light–dark regime (lights on at 7:00 am). Food and water were available ad libitum. Further details of the health, number of rats used for each of the studies, the weights and injected doses are provided in Supplementary Table 2.

This research was regulated under the Animals (Scientific Procedures) Act 1986 Amendment Regulations 2012 following ethical review by the University of Cambridge Animal Welfare and Ethical Review Body (AWERB).

### PET Scans

Rats were anaesthetised using isoflurane and femoral vein and artery of each rat was cannulated, as previously described [21] for the injection of the radiotracer and blood sampling respectively. Individual rats were placed prone on the warmed bed of the PET-CT scanner (Mediso nanoScan PET-CT, Hungary) and physiologically monitored during the scans.

A CT scan (helical acquisition, 360 projections, 50 kVp/980uA tube voltage, 300 ms exposure time and 1:4 binning) was performed and images were reconstructed with Cosine Filter Back Projection method. [^18^F]AV1451 was injected over ~ 20 s via the femoral vein cannula and a 60 min PET scan (packet timestamp list-mode, single field-of-view, 1–5 coincidence mode, 5 ns coincidence time window) of the head started simultaneously. The list mode data were re-binned in 22 time frames (6 × 10 s, 4 × 30 s, 4 × 60 s, 1 × 180 s, 4 × 300 s, 3 × 600 s) and dynamically reconstructed (Tera-Tomo 3-dimensional reconstruction [22] with 4 iterations and 6 subsets; 400–600 keV; 0.4 mm^3^ voxel size). Data were corrected for decay, attenuation, scatter and random coincidences.

Sixteen arterial blood samples were collected and placed on ice at approximately 5, 10, 15, 30, 45, 60, 75, 90, 105 s (~ 70 uL) and 2, 3, 5, 10, 15, 30 and 60 min (~ 150 uL) post injection. A 25 μL aliquot of whole blood was reserved, and remainder centrifuged (Eppendorf 5430-R centrifuge, at 5 °C, 5 min, 30,130 RCF) to obtain plasma. Radioactivity in plasma and whole blood (25 μL each) was measured using a gamma counter (Hidex AMG 425–601). Whole blood and plasma time-activity curves (TACs) were plotted and the results expressed as %ID/g. Remaining plasma samples (5, 10, 15, 30 and 60 min) were further processed (see supplementary information) for metabolite analysis. The parent radioligand concentration was expressed as a percentage of the total plasma radioactivity.

At the end of the scan, the animals were culled whilst under deep anaesthesia, brain dissected out, bisected along the sagittal plane, frozen over dry ice and stored at −80 °C. A biodistribution study was performed in six rats from each group (see supplementary information).

### Kinetic Modelling of PET Data

**Fig. 1:**
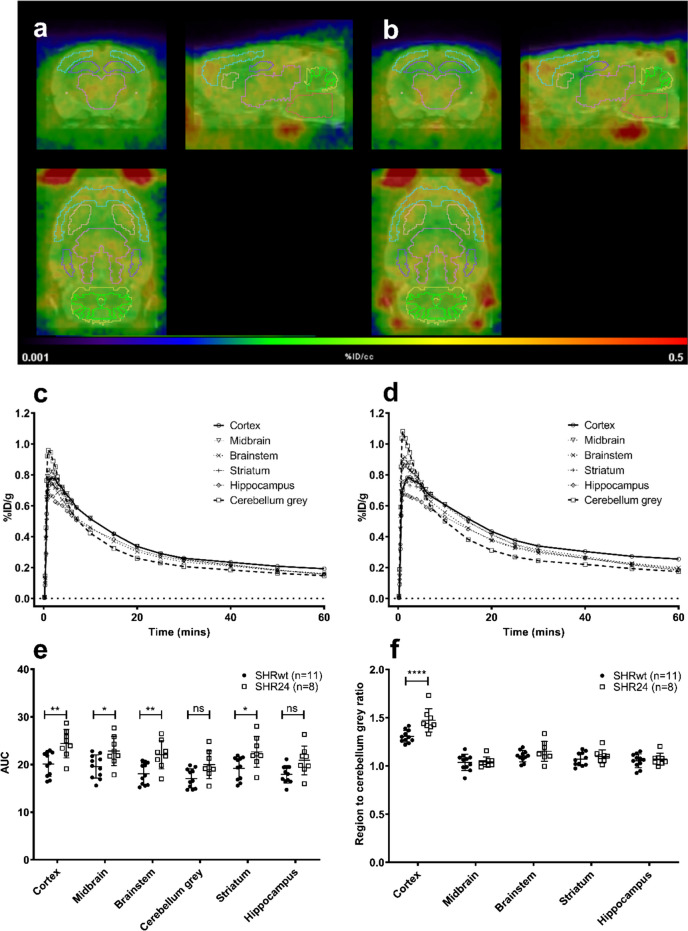
Representative PET scan images (%ID/g, 0–60 min summed) of [^18^F]AV1451 uptake in the brains of (**a**) SHRwt and (**b**) SHR24 rats co-registered with an MRI template and ROIs visualised: cortex (blue), midbrain (pink), brainstem (red), striatum (orange), hippocampus (purple), cerebellum grey (yellow) and cerebellum white (green). Average TACs (without error bars for visual clarity) of the brain regions of (**c**) SHRwt and (**d**) SHR24 show higher variability in uptake between regions in SHR24 rats. (**e**) Area under the curves of the groups show higher overall uptake in the brains of SHR24 rats. (**f**) Region to cerebellum grey ratio of 50–60 min post injection reveals that only the cortex has significantly higher uptake

Post processing and kinetic modelling of the PET data was performed using PMOD software (v3.8; PMOD technologies, Zurich). CT images from individual rats were co-registered (rigid match) with an MR template (Schiffer T2 rat). The transformations were applied to individual PET scans and manually adjusted where required. 3D volumes of interest (VOIs) available on PMOD for the MR template (Px Rat W.Schiffer) were modified (see supplementary information) to obtain cortex, midbrain, brainstem, cerebellar grey, striatum and hippocampus VOIs (Fig. 1a and b). TACs were obtained for each of the VOIs and the results expressed as %ID/g. Area under the TACs were obtained (Graphpad Prism, version 9.4.1). Additionally, the ratio between the region and cerebellar grey was calculated and differences between groups analysed using 2-way ANOVA followed by post hoc Bonferroni test.

Two-tissue compartment model (2-TCM), with the parameter for cerebral blood volume (V_B_) fixed at 3.6% [23], was fitted to the dynamic PET data of brain regions using PMOD software. This model was chosen following comparison of various models (1-TCM with V_B_ free, 1-TCM with V_B_ fixed at 3.6%, 2-TCM with V_B_ free, 2-TCM with V_B_ fixed at 3.6% and 2-TCM with V_B_ fixed at 3.6% with added blood delay) via visual comparison and using Akaike information criterion values during preliminary analysis of the whole brain VOI. Blood and plasma TACs were used with linear interpolation. Group population average metabolite curve fractions were used for correcting individual plasma curves. The rate constants K_1_, k_2_, k_3_ and k_4_ were estimated from the curve fit and macro parameters, non-displaceable volume of distribution (V_ND_ = K_1_/k_2_), total volume of distribution (V_T_ = K_1_/k_2_ * (1 + k_3_/k_4_)) and non-displaceable binding potential (BP_ND_ = k_3_/k_4_) were obtained.

Additionally, Logan graphical analysis (t* = 20, V_B_ = 3.6%) was also used to estimate V_T_ (V_T_ Logan). To evaluate the possibility of using this model in longitudinal studies without blood sampling, Simplified reference tissue model (SRTM) and Logan reference tissue model (LRTM) were fitted using cerebellum grey as the pseudo-reference region. The cerebellum was chosen because it does not exhibit hyperphosphorylated tau or tau pathology at any age. The constant $\mathrm{k_2^\prime}$ from SRTM was used for LRTM. The correlation between V_T_ Logan and V_T_ and between k_3_/k_4_ and BP_ND_ from the reference tissue models were quantified using Pearson correlation coefficient r. The predictive value of V_T_ Logan for V_T_ and BP_ND_ from reference tissue models for k_3_/k_4_ from 2-TCM were evaluated using regression analysis.

The primary outcome measures were differences in V_T_ and BP_ND_ from the cortex. Statistical analyses (t-test and 2-way ANOVA with Bonferroni correction for multiple comparison) were carried out using GraphPad Prism (v 9.1.2 and 9.4.1). Significance levels are denoted as *P < 0.05, ** P < 0.01, *** P < 0.001 and **** P < 0.0001.

### *In Vitro* Studies

Fresh frozen sagittal sections (30 μm) of the brain stored at −80 °C was used for autoradiography [24]. The sections were fixed in ice cold methanol (20 min) and incubated with 0.85 MBq/ml [^18^F]AV1451 in phosphate buffered saline (PBS) at 37 °C (1 h). Adjacent slides were similarly incubated with [^18^F]AV1451 in the presence of 10 μM [^19^F]AV1451. Slides were washed as follows: ice-cold PBS (1 min), 70% ethanol/30% PBS (2 min), 30% ethanol/70% PBS (1 min), ice-cold PBS (1 min) and dipped into ice-cold de-ionised water (1 s). The slides were dried in a stream of air and exposed to a phosphor-imaging plate (GE Healthcare) overnight. The plates were read on a phosphorimager (Dürr Medical CR35 Bio Digital Imaging System) and analysed using AIDA Image Analyser (v.4.27). Regions of interest were drawn on cortex, midbrain, brainstem, cerebellum (pseudo-reference region) and parietal/retrosplenial cortex (low uptake region) (Fig. 6c). [^18^F]AV1451 binding data from each region is expressed as a ratio to the binding on individual cerebellum. Statistical analyses of all autoradiography results were carried out with two-way repeated measure ANOVA with Bonferroni multiple-comparison correction. Statistical levels are denoted as *P < 0.05, ** P < 0.01, *** P < 0.001 and **** P < 0.0001. Five representative slides from the autoradiography study were stored at −80 °C until immunofluorescence and fluorescence staining experiments (see supplementary information).

## Results

### Radiochemistry

The radiosynthesis yielded [^18^F]AV1451 with 100% radiochemical purity in good yields and molar activity (31.08 ± 7.41 GBq.µmol^−1^, *n* = 15) at the end of synthesis (see Supplementary Table 1).

### Animals

Details of the number of rats used for each of the studies, the weights and injected doses are provided in Supplementary Table 2. SHR24 rats were of significantly lower weight (261.0 ± 5.3, *n* = 8 vs 392.2 ± 9.4, *n* = 11, *P* < 0.0001) compared to SHRwt rats due to loss of fat and muscle mass by this age.

### PET Scans and TACs

PET scans and TACs of the brain regions indicated peak uptake within the first three minutes in both groups, followed by slower washout in SHR24 rats. At the end of the 60-min scan, the highest uptake was seen in the cortex and the lowest in the cerebellar grey. There were larger regional differences in uptake in SHR24 rats compared to SHRwt rats (Figs. 1a-f). When the Area under the curves (AUC) of the TACs were compared (Fig. 1e), SHR24 rats had significantly higher uptake in all brain regions except cerebellar grey and hippocampus after correcting for multiple comparison. However, significant difference was observed only in the cortex when the region to cerebellum ratios were compared at the last time point (Fig. 1f and Supplementary Fig. 1a). An increase in bone uptake was observed in both groups as the scan progressed (Supplementary Fig. 1b, c).

**Fig. 2:**
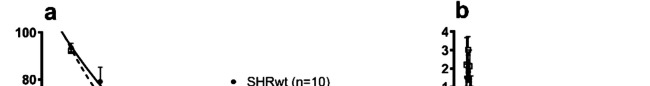
(**a**) Percentage of intact [^18^F]AV1451 remaining in the plasma over time in the two groups and (**b**) metabolite-corrected plasma TACs of [^18^F]AV1451 in the two groups. One SHRwt rat is not included in the plasma graph as blood sampling was not at standard time points

Plasma metabolite analysis revealed that both groups metabolised [^18^F]AV1451 to a similar extent with 18% parent remaining at 60 min in SHRwt rats, compared to 15% in SHR24 rats (Fig. 2a). AUC was however significantly higher (2385 ± 350 vs 2064 ± 253, *P* < 0.05) in SHRwt rats. Metabolite-corrected plasma TACs were monoexponential for both groups, peaked around 30 s and was followed by rapid washout with no apparent group differences. However, plasma exposure of the intact [^18^F]AV1451, calculated as AUC of the metabolite-corrected plasma TAC, was significantly higher (5.6 ± 0.7 vs 4.8 ± 0.7, *P* < 0.05) in SHR24 rats compared to SHR rats (Supplementary Fig. 2).

### Kinetic Modelling of PET Data

**Fig. 3:**
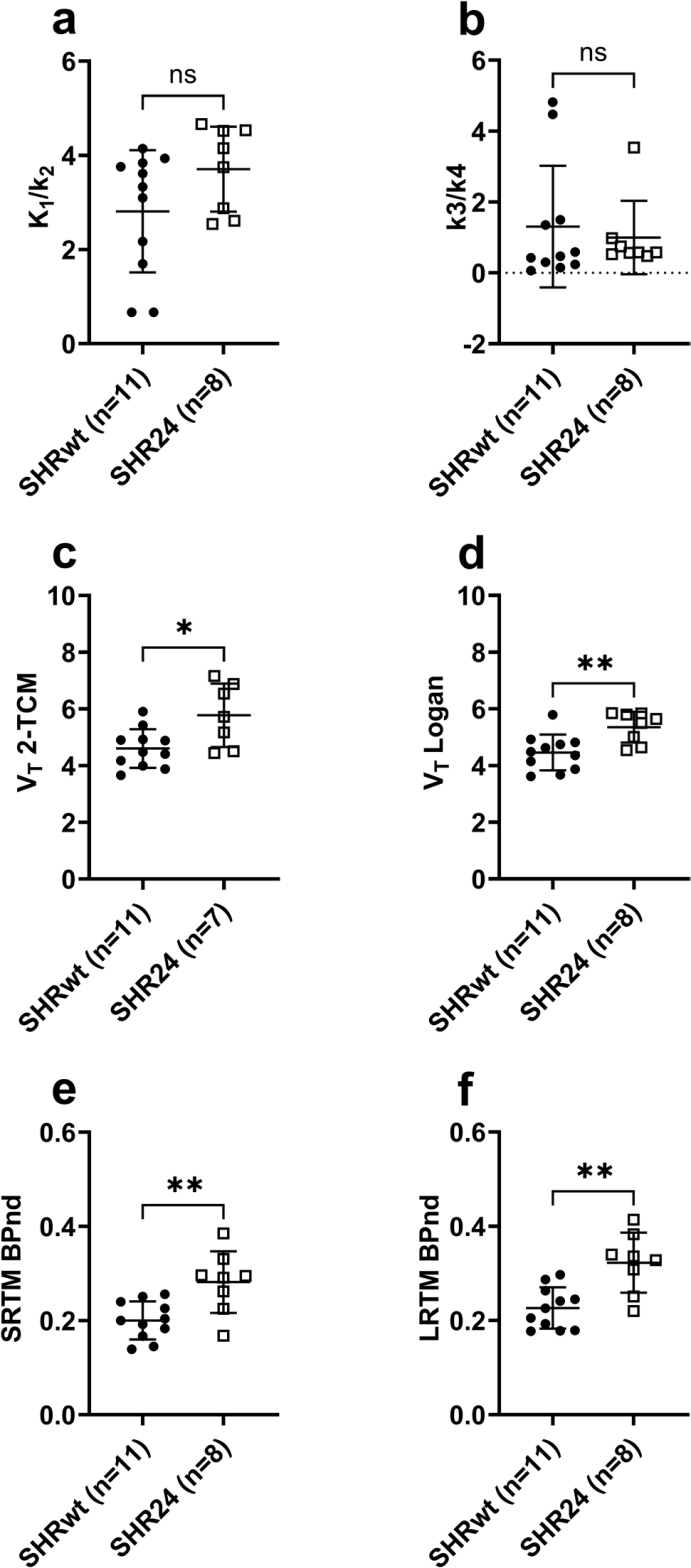
Kinetic modelling parameters from cortex. (**a**) K_1_/k_2_ and **(b)** binding potential, k_3_/k_4_, estimated from 2-TCM were highly variable. Total distribution volume (V_T_) estimated from (**c**) 2-TCM and (**d**) Logan graphical analysis yielded significantly higher values in SHR24 rats. BP_ND_ from the (**e**) Simplified Reference tissue Model (SRTM) and (**f**) Logan Reference Tissue Model (LRTM), were less variable compared to that from 2-TCM and revealed significantly higher values in SHR24 rats. One SHR24 animal was excluded from the analysis of V_T_ from 2-TCM as an outlier (V_T_ = 20.5)

In the cortex, K_1_/k_2_ (Fig. 3a) and binding potential (k_3_/k_4_) (Fig. 3b) values exhibited considerable variability with no significant differences observed between the groups. Significantly higher V_T_ values (5.8 ± 1.1 vs 4.6 ± 0.7, *P* < 0.05) were estimated using 2-TCM in SHR24 rats compared to SHRwt rats (Fig. 3c). V_T_ estimated from Logan graphical analysis also revealed significantly higher values in SHR24 rats (Fig. 3d) (5.3 ± 0.5 vs 4.5 ± 0.6, *P* < 0.01) and had lower variability. BP_ND_ values obtained from SRTM (0.28 ± 0.07 vs 0.20 ± 0.04, *P* < 0.01) and LRTM (0.32 ± 0.06 vs 0.23 ± 0.04, *P* < 0.01) were both significantly higher in SHR24 rats compared to SHRwt rats (Fig. 3e, f). Defining k_3_/k_4_ values above one as outliers revealed apparent significant differences between SHR24 and SHRwt rats (Supplementary Fig. 3).

**Fig. 4:**
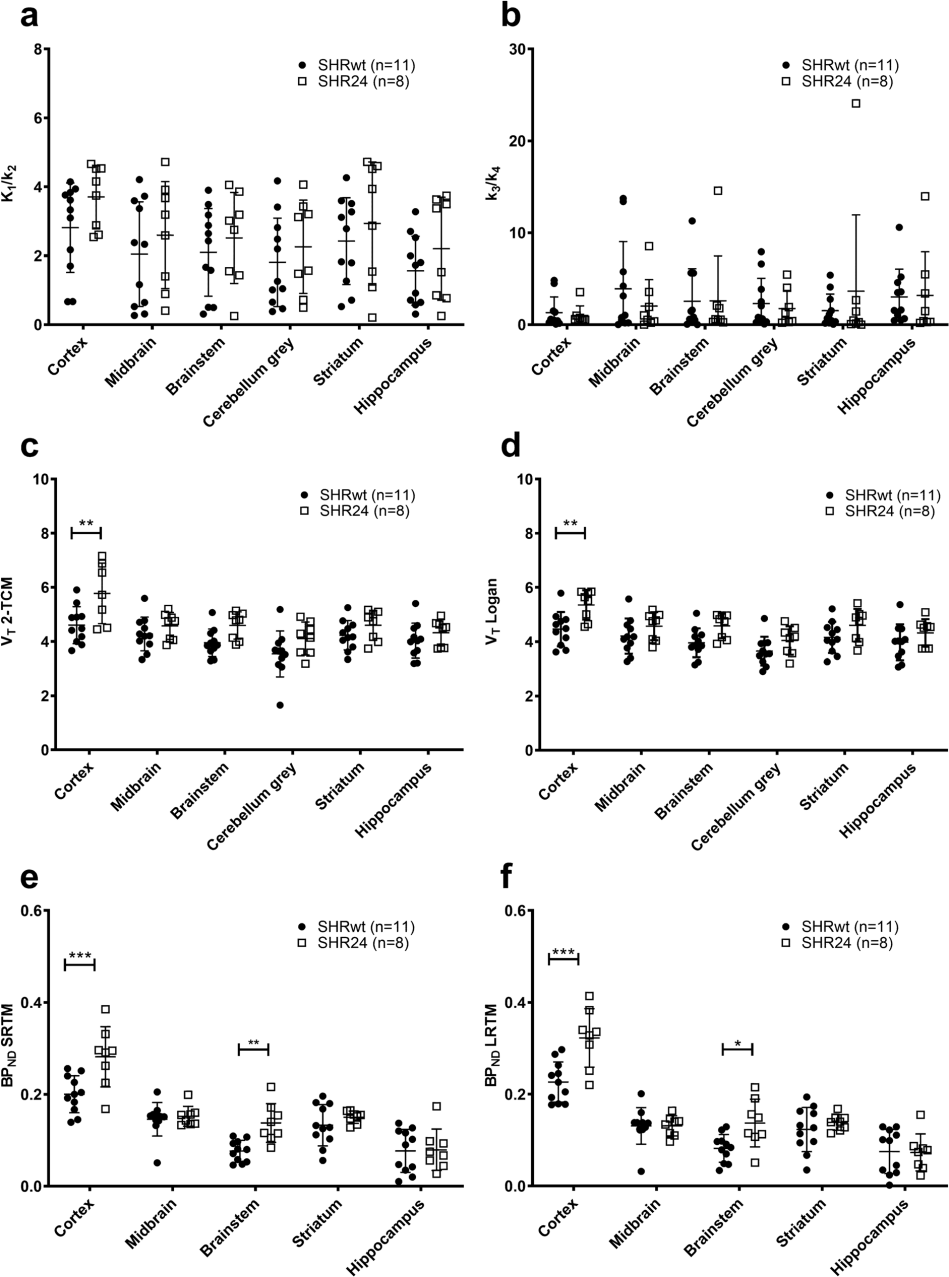
Regional kinetic modelling parameters. (**a**) K_1_/k_2_ and (**b**) binding potential, k_3_/k_4_, estimated from 2-TCM were highly variable in all regions. Total distribution volume (V_T_) estimated from (**c**) 2-TCM and (**d**) Logan graphical analysis, showed significantly higher values only in the cortex of SHR24 rats. While binding potential obtained from 2-TCM was highly variable, BP_ND_ from the (**e**) Simplified Reference tissue Model (SRTM) and (**f**) Logan Reference Tissue Model (LRTM) were less variable and revealed significantly higher values in SHR24 rats in the cortex and brainstem. One SHR24 animal’s cortex was excluded from the analysis of V_T_ from 2-TCM as an outlier (V_T_ = 20.5)

Similar to the cortex, K_1_/k_2_ (Fig. 4a) from the rest of the brain regions displayed high variability with no significant differences observed between the groups. The binding potential (k_3_/k_4_) (Fig. 4b) values were also highly variable with multiple ‘outliers’ and significant differences were again not observed. V_T_ estimates from both 2-TCM (Fig. 4c) and Logan graphical analysis (Fig. 4d) revealed significant effect of group and brain regions, without interaction between them. After correcting for multiple comparisons, only cortex exhibited significantly higher V_T_ values (5.8 ± 1.1 vs 4.6 ± 0.7, *P* < 0.01) in SHR24 rats compared to SHRwt rats. BP_ND_ values from SRTM (Fig. 4e) and LRTM (Fig. 4f) displayed similar patterns with significant effect of group and brain regions as well as interaction between them. Significantly higher binding of [^18^F]AV1451 was observed in the cortex (0.28 ± 0.07 vs 0.20 ± 0.04, *P* < 0.001, by SRTM) and brainstem (0.14 ± 0.04 vs 0.08 ± 0.02, *P* < 0.01, by SRTM) after correction for multiple comparisons.

**Fig. 5:**
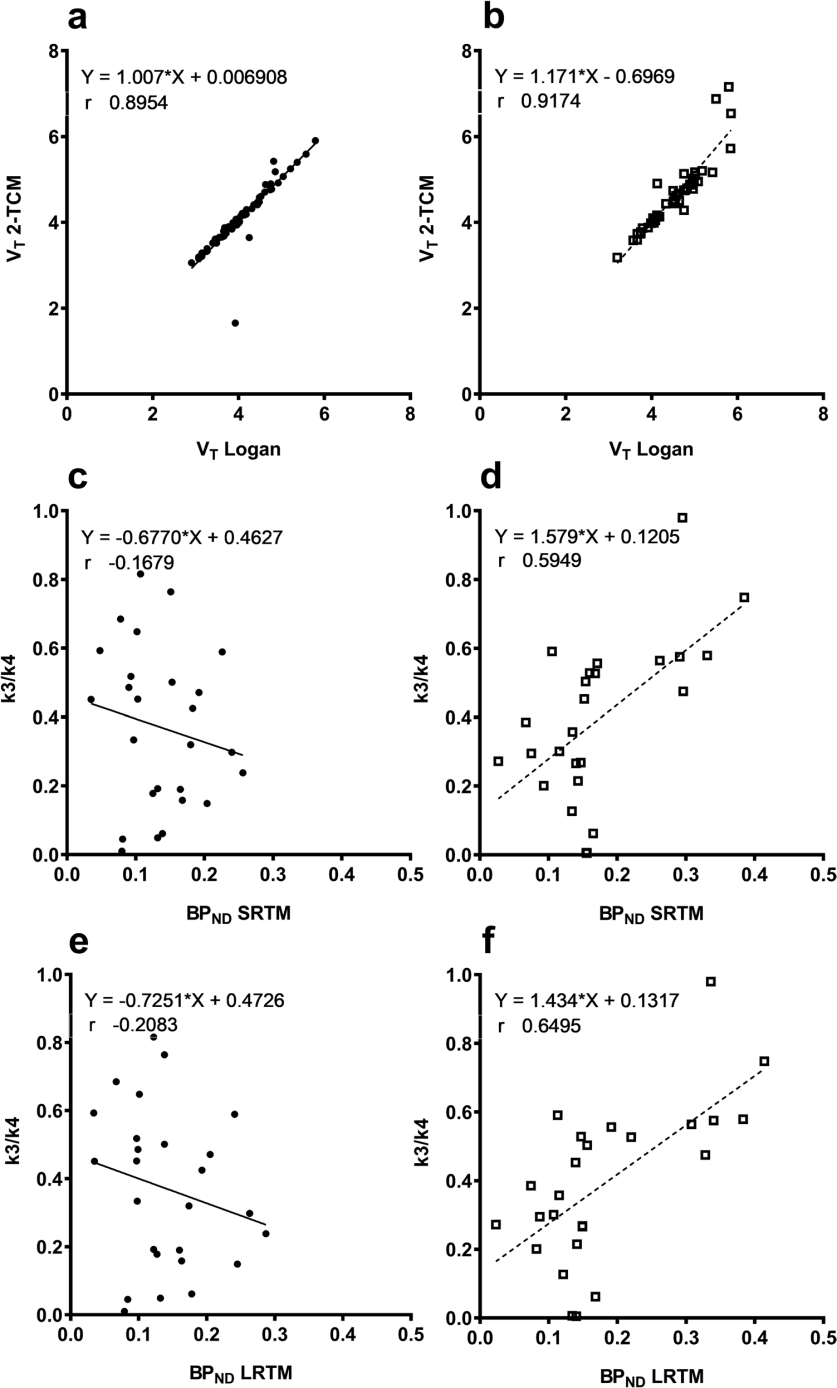
Correlation and regression analysis of parameters from 2-TCM with V_T_ Logan graphical analysis and BP_ND_ from reference tissue models in (**a, c, e**) SHRwt and (**b, d, f**) SHR24 rats. V_T_ from Logan graphical analysis and 2-TCM were highly correlated in both (**a**) SHRwt (66 data points) and (**b**) SHR24 rats (47 data points, one outlier from 2-TCM = 20.5 excluded). While BP_ND_ from reference tissue models poorly correlated with k_3_/k_4_ from 2-TCM in (**c, SRTM** and **e, LRTM**) SHRwt rats (26 data points), moderate positive correlations were observed in (**d, SRTM** and **f, LRTM**) SHR24 rats (25 data points)

V_T_ from Logan graphical analysis and 2-TCM were highly correlated (Pearson r = 0.9) in both SHRwt and SHR24 rats (Fig. 5a and b). However V_T_ Logan underestimated V_T_ by 17% (y = 1.171 * x – 0.6969) in SHR24 rats. Due to the high variability of k_3_/k_4_ from 2-TCM, only those values below one were considered for correlation and regression analysis. In SHRwt rats, BP_ND_ from the reference tissue models were poorly correlated with k_3_/k_4_ (Fig. 5c and e). In SHR24 rats (Fig. 5d and f) there were moderate positive correlations (Pearson r = 0.59 for SRTM and 0.6 for LRTM). SRTM underestimated BP_ND_ by 58% (y = 1.579 * x + 0.1205) while LRTM underestimated BP_ND_ by 43% (y = 1.434 * x + 0.1317) in SHR24 rats.

### *In Vitro* Studies

**Fig. 6:**
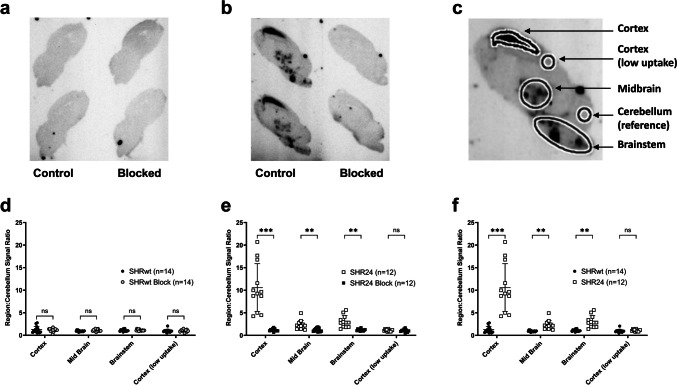
Autoradiography. Representative autoradiography images of [^18^F]AV1451 in (**a**) SHRwt and (**b**) SHR24 rats. Higher binding of [^18^F]AV1451 is seen in the cortex, midbrain and brainstem regions of SHR24 rats which was blocked by 10uM [^19^F]AV1451 whereas there was negligible binding in SHRwt rats which did not change considerably with blocking (right-hand slides). (**c**) displays the various brain regions of interest that were analysed. 2-way ANOVA followed by Bonferroni analysis of the autoradiography images revealed (**d**) insignificant blocking in SHRwt rats, (**e**) significant blocking in SHR24 rats and (**f**) significantly higher binding of [^18^F]AV1451 in the cortex, midbrain and brainstem regions of SHR24 rats compared to SHRwt rats

Autoradiography images revealed negligible binding in SHRwt rats (Fig. 6a). In SHR24 rats (Fig. 6b), binding was evident in the cortex, midbrain and brainstem regions as confirmed by fluorescence imaging (Supplementary Fig. 5). 2-way ANOVA followed by Bonferroni analysis of the various brain regions revealed no significant blocking of [^18^F]AV1451 by 10uM [^19^F]AV1451 in SHRwt rats (Fig. 6d) and significant blocking in SHR24 rats (Fig. 6e). When comparing SHR24 and SHRwt rats, significant higher binding of [^18^F]AV1451 was observed in the brain regions (Fig. 6f), with the highest difference observed in the cortex (10.6 ± 5.3 vs 1.3 ± 0.7, *P* < 0.001) and moderate differences in the midbrain (2.28 ± 1.02 vs 0.94 ± 0.15, *P* < 0.01) and brainstem (3.1 ± 1.3 vs 1.1 ± 0.3, *P* < 0.01).

## Discussion

Herein, we evaluated the kinetics of [^18^F]AV1451 in SHR24 rat brain expressing human truncated tau, with the intention of utilizing this model in future PET studies on aggregated tau neurobiology and for the evaluation of novel tau radioligands. In vivo evaluation of novel PET radioligands for tau in transgenic mice is challenging due to the small brain size and blood volume, making full kinetic evaluation nonviable. SUV values are often used as binding potential surrogates, which is not suitable in situations [25] where weight loss occurs in the transgenic animals compared to healthy wildtype controls as well as when SUV has not been validated against gold-standard blood based kinetics. This validation is essential, as SUV as a measure of binding overlooks rate differences in tracer delivery, uptake into brain, metabolism or clearance between groups.

Loss of fat and muscle in SHR24 rats led to significant weight loss by 13–15 months. Therefore, SUV became unreliable for [^18^F]AV1451, as heavier animals exhibit higher SUVs [25] due to correction for weight when the radiotracer has limited distribution into fat and muscle (Supplementary Table 2). On the other hand, as %ID/g does not account for weight differences, SHR24 rats exhibited higher uptake across multiple regions not expected to have high uptake. To overcome this bias, the region-to-cerebellum ratios (50–60 min p.i.) (Fig. 1f) were compared which resulted in cortex alone displaying significantly higher values in SHR24 rats compared to SHRwt rats. Higher cortical uptake compared to other regions was also seen in SHRwt rats. The presence of inflammatory markers [26], astrogliosis and blood–brain barrier disruption [27] in the cortex of SHR rats implies potential presence of MAO-B [28] or blood components to which [^18^F]AV1451 is known to have off-target binding [3, 29]. Considering the impact of weight differences, cross-validation with kinetic modelling is required before application of SUV, %ID/g or their ratios.

While the AUC of metabolite curve and metabolite-corrected plasma curves (Supplementary Figs. 2b and d) were statistically different, the differences were small and unlikely to be of biological significance. As reported by others, a progressive increasing bone uptake, potentially due to defluorination, was observed in these rats [30].

Estimation of V_T_ from 2-TCM and Logan graphical analysis yielded comparable results with high correlation, with both approaches exhibiting significantly higher values in the cortex. However, binding potential (k_3_/k_4_) was highly variable and poorly estimated from 2-TCM. To assess the correlation between k_3_/k_4_ and BP_ND_ from the more stable reference tissue models, a cut-off value of one was imposed for the more variable k_3_/k_4_. This value was chosen based on visual observation of the distribution of data and comparison to human data [31] where maximum k_3_/k_4_ values were around 2 while maximum V_T_ values were around 16, which is half of what was observed in our study. Consequently, over 50% of the 2-TCM derived k_3_/k_4_ data was discarded. Although reference tissue models provided data that was less variable, they under estimated BP_ND_. The underestimation of BP_ND_ from reference tissue models should be interpreted in the context of the potential inaccurate estimation of k_3_/k_4_ from 2-TCM. While V_T_ (Logan) can be used instead of V_T_ from 2-TCM, it cannot be used instead of BP_ND_, as the correlation was weak (Supplementary Fig. 4) and the significant difference observed in brainstem BP_ND_ is not seen in V_T_ (Logan). The higher BP_ND_ observed in cortex and brainstem aligns with observations from the autoradiography study.

In human PET studies of [^18^F]AV1451 on AD patients (3R + 4R tau), the highest V_T_ from 2-TCM in the most affected regions were in the region of 14–16, while BP_ND_ from 2-TCM and reference tissue models were in the range of 2–2.5 [31]. In non-AD pathologies like frontotemporal dementia (3R tau or 4R tau) [32] and progressive supranuclear palsy (4R tau) [33], highest reference tissue model derived BP_ND_ was lower at 1.15 and 0.6 respectively, while 2-TCM data has not been published. Based on the human data, the highest values obtained from our study V_T_ (about 7) and BP_ND_ (about 0.4 from SRTM) are within the expected range for [^18^F]AV1451.

While the binding of [^18^F]AV1451 appeared highly specific in the autoradiography study with near complete blocking when treated with [^19^F]AV1451, it should be noted that the autoradiography protocol we performed involved washes in buffer containing ethanol. Without such washing steps, the known regional distribution of tau in SHR24 rats were not visualised (Supplementary Fig. 6). This suggests that ethanol containing buffer wash is likely to have washed away weak or off-target bound [^18^F]AV1451 implying that off target binding of AV1451 could be contributing to the observed scan data.

We have established that the binding of [^18^F]AV1451 to NFTs in this rat model could be detected by dynamic PET scans and kinetic modelling, allowing us to quantify the tau load. Despite the known off-target binding and defluorination of [^18^F]AV1451, we were able to demonstrate increased binding of the radiotracer in the brain regions with known NFT accumulation in the SHR24 rat model. With the ability to perform full kinetic modelling, we have demonstrated the utility of this model as translational platform for assessing aggregated tau neurobiology by PET, including for the evaluation of tau PET radioligands. Use of reference tissue models will additionally allow longitudinal scans to be performed without the need for blood sampling.

## Conclusion

In this study, we aimed to validate the use of a rat model expressing human truncated tau as a translational platform for investigating aggregated tau neurobiology by PET. A rat model is desirable over a mouse model due to practicalities of performing blood-based gold standard kinetic modelling before validating and applying simplified quantification methods. We demonstrated that V_T_, from 2-TCM and Logan graphical analysis, identified cortex as the region with the highest tau accumulation. Additionally, BP_ND_ from SRTM identified an additional region, brainstem. This study therefore validates the SHR24 rat model as a translational platform for assessing aggregated tau neurobiology by PET, including evaluating the binding properties of novel radioligands to NFTs. Additionally, validating reference tissue models also provides an opportunity to follow changes in tau levels longitudinally within the same animal.

## Supplementary Information

Below is the link to the electronic supplementary material.

Supplementary file1 (DOCX 13584 KB)

## Data Availability

The data are available from the corresponding author on reasonable request.
