# Supplementary material for: PET Imaging of a Transgenic Tau Rat Model SHR24 with [18F]AV1451
Source: Mol Imaging Biol. 2025 Jan 21;27(2):238–49. doi: 10.1007/s11307-024-01972-4 (PMC12062146; doi:10.1007/s11307-024-01972-4)

**PET imaging of a transgenic tau rat model SHR24 with [^18^F]AV1451**

Author Information

Nisha K. Ramakrishnan^1,2^, Annie Ziyi Zhao^1^, Stephen Thompson^1^, Selena Milicevic Sephton^1^, David J. Williamson^1^, Tomáš Smolek^3,4^, Norbert Žilka^3,4^, Franklin I. Aigbirhio^1^

1. Molecular Imaging Chemistry Laboratory, Wolfson Brain Imaging Centre, Department of Clinical Neurosciences, University of Cambridge, Cambridge CB2 0QQ, United Kingdom

2. Preclinical Imaging Facility, Anne McLaren Building, 90 Francis Crick Ave, Trumpington, Cambridge CB2 0BA, United Kingdom

3. Axon Neuroscience R&D Services SE, Dubravska vćesta 9, 811 02 Bratislava, Slovakia

4. Institute of Neuroimmunology, Slovak Academy of Sciences, Dubravska cesta 9, 845 10 Bratislava, Slovakia

**Radiochemistry**

The radiosynthesis of [18F]AV1451 was achieved in two steps following the previously published protocol by the Scott group[20]. Briefly, the radiosynthesis was automated and conducted on the FXFN radiosynthesis module. The radiolabelling precursor was Boc-protected 2-nitropyridyl analogue which was added as 0.5 mg in 0.5 mL anhydrous DMSO to dried fluorine-18. Fluorine-18 was trapped on the QMA light carbonated cartridge and eluted with 7 mg/mL aqueous K2CO3. Then 15 mg/mL ethanolic K222 was added and material azeotropically dried. The crude product was purified by semi-preparative HPLC (eluting with 40% EtOH, 60% 10 mM NaH2PO4, pH 9.3), then eluted with EtOH from a conditioned HLB 1 cc, 10 mg cartridge and formulated as a 0.9% saline solution.

The radiosynthesis yielded [18F]AV1451 with 100% radiochemical purity in good yields and molar activity (31.08 ± 7.41 GBq.µmol-1, n=15) at the end of synthesis (Supplementary Table 1). We have observed a fairly large variation in decay corrected radiochemical yields amongst different batches of [18F]AV1451 which correlated with the amount of starting fluorine-18 whereby smaller amount of starting radioactivity resulted in higher decay corrected radioactive yields.

**Supplementary table 1:** Table summary of radiochemistry syntheses of various batches of [^18^F]AV1451.


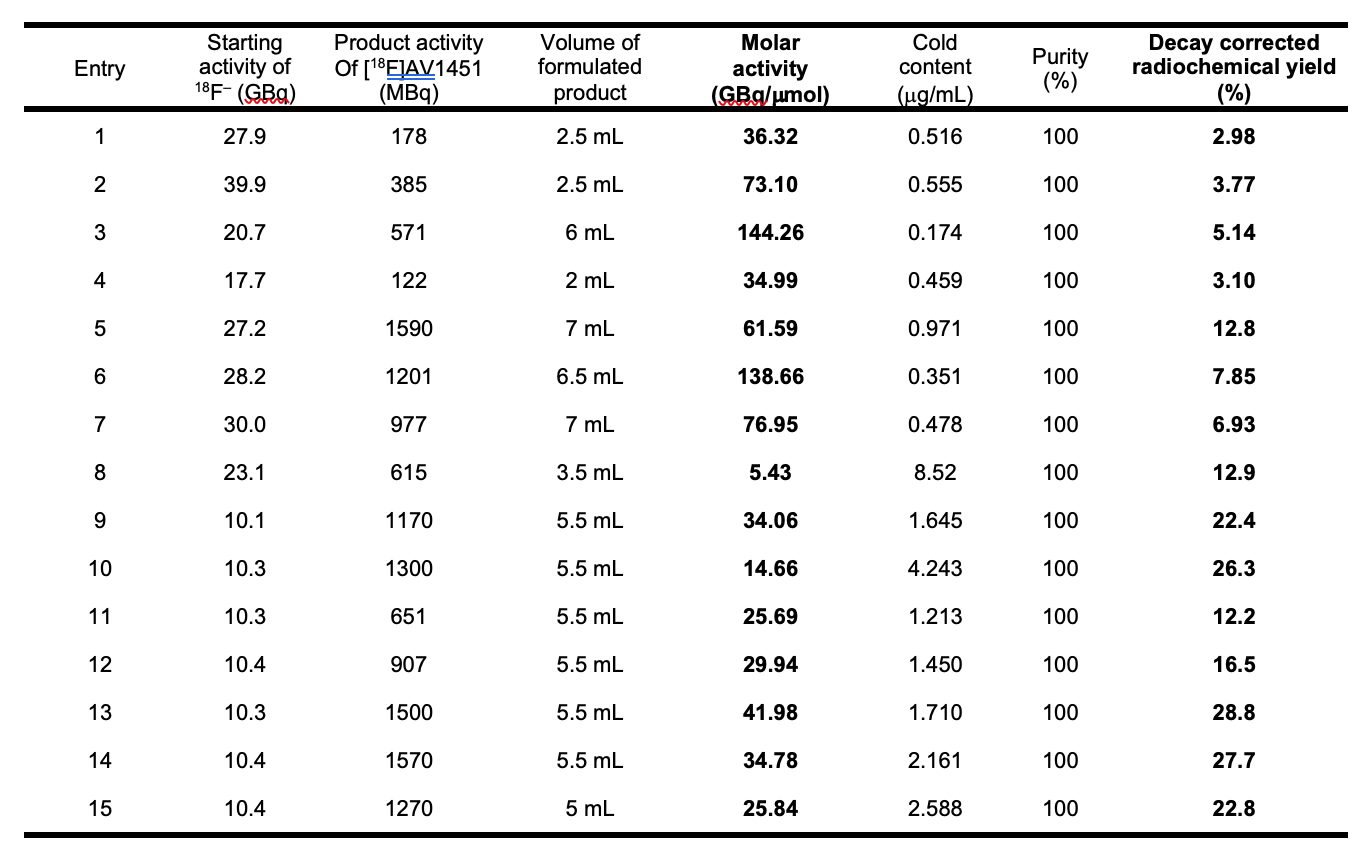


**Details of animals used in this study (Mean** **± SEM)**

Male SHR24 transgenic tau rats (SHR24) (n = 13) and their age-matched wildtype SHR littermates (SHRwt) (n = 15) were imported from Axon Neuroscience at 13-14 months old. They were allowed to acclimatise for a minimum of seven days before performing non-recovery scans over the next 2 months. They were monitored using a traffic light scoring system- their impairments were at the amber stage (hind limb weakness and reduced mobility, beginning to lose weight, beginning to lose muscle tone on hind limbs) at the point of importing and the humane endpoint was the red stage (if they are not able to move about the cage, has respiratory impairment, or rapid weight loss approaching 15%). One each of SHR24 and SHRwt rat were removed from the study before the start of the scans due to deterioration in health approaching humane endpoint.

Nine SHR24 and eleven SHRwt rats underwent dynamic PET scans. One SHR24 rat was removed from the analysis due to malfunction of the venous cannula. Three rats from each group were part of a pilot study (on an older scanner) and only metabolite, biodistribution and autoradiography data from these animals were included in this manuscript.

Randomisation and Blinding: Randomisation was not performed. Where possible one rat from each group were scanned on each day, however due to the deterioration of the health of some of the SHR24 rats, they had to be prioritised for scanning. Blinding was not performed.

**Supplementary table 2:**

| **Experimental process** | **SHRwt** | **SHR24** |
| --- | --- | --- |
| Total number of animals in the study | 14 | 12 |
| Pilot study (not included in kinetic modelling) | 3 | 3 |
| Kinetic modelling | 11 | 8 |
| Scanned animals not included in kinetic modelling |  | 1 |
| Arterial input function | 11 | 8 |
| Biodistribution (includes animals from pilot study) | 6 | 6 |
| Metabolite analysis (includes animals from pilot study) | 10 | 9 |
| Autoradiography (includes animals from pilot study) | 14 | 12 |

|  | **SHRwt (n=11)** | | | **SHR24 (n=8)** | | | ***P*** |
| --- | --- | --- | --- | --- | --- | --- | --- |
| **Weight (g)** | 392.4 | ± | 9.4 | 261.0 | ± | 5.3 | <0.0001 |
| **Injected dose (MBq)** | 33.68 | ± | 2.0 | 31.94 | ± | 1.4 | ns |

**Biodistribution**

A biodistribution was performed in a few (n = 6) rats from each group where blood, various brain regions (from half of the brain) and peripheral tissues were dissected out and the radioactivity in the samples was measured and the samples weighed using a gamma counter (Hidex AMG 425-601). The results were expressed as %ID/g or dimensionless standardised uptake values (SUV = [(tissue activity concentration) x (body weight)]/injected dose). SUVs were calculated assuming a specific gravity of 1 g.mL-1 for brain tissue.

**Supplementary table 3:** Biodistribution data (SUV) at 60 min post injection of [^18^F]AV1451 (Mean ± SEM). Differences between groups were examined separately for brain regions and peripheral tissues using 2-way ANOVA followed by post hoc Bonferroni test.

|  | **%ID/g** | | | | | | **SUV** | | | | | |
| --- | --- | --- | --- | --- | --- | --- | --- | --- | --- | --- | --- | --- |
| **Tissue** | **SHRwt (n=6)** | | | **SHR24 (n=6)** | | | **SHRwt (n=6)** | | | **SHR24 (n=6)** | | |
| **Brain** | | | | | | | | | | | | |
| Bulbus | 0.151 | ± | 0.011 | 0.137 | ± | 0.010 | 0.586 | ± | 0.039 | 0.383 | ± | 0.024** |
| Hippocampus | 0.147 | ± | 0.013 | 0.145 | ± | 0.014 | 0.570 | ± | 0.048 | 0.405 | ± | 0.034* |
| Striatum | 0.145 | ± | 0.014 | 0.124 | ± | 0.010 | 0.558 | ± | 0.055 | 0.343 | ± | 0.023** |
| Cortex | 0.157 | ± | 0.014 | 0.159 | ± | 0.016 | 0.611 | ± | 0.050 | 0.443 | ± | 0.038* |
| Cerebellum | 0.129 | ± | 0.011 | 0.125 | ± | 0.012 | 0.503 | ± | 0.042 | 0.346 | ± | 0.028* |
| Pons and Medulla | 0.155 | ± | 0.013 | 0.164 | ± | 0.014 | 0.601 | ± | 0.049 | 0.456 | ± | 0.032 |
| Rest of brain | 0.155 | ± | 0.013 | 0.155 | ± | 0.014 | 0.600 | ± | 0.049 | 0.431 | ± | 0.032* |
|  | | | | | | | | | | | | |
| **Periphery** | | | | | | | | | | | | |
| Pituitary | 0.375 | ± | 0.034 | 0.425 | ± | 0.039 | 1.463 | ± | 0.252 | 1.185 | ± | 0.090 |
| Submandibular gland | 1.161 | ± | 0.052 | 1.188 | ± | 0.074 | 4.528 | ± | 0.167 | 3.320 | ± | 0.185 |
| Lung | 0.730 | ± | 0.044 | 0.838 | ± | 0.044 | 2.838 | ± | 0.172 | 2.345 | ± | 0.113 |
| Heart | 0.457 | ± | 0.046 | 0.447 | ± | 0.038 | 1.776 | ± | 0.283 | 1.251 | ± | 0.101 |
| Liver | 2.205 | ± | 0.076 | 2.220 | ± | 0.065 | 8.573 | ± | 0.122 | 6.210 | ± | 0.134** |
| Pancreas | 0.390 | ± | 0.027 | 0.427 | ± | 0.060 | 1.525 | ± | 0.559 | 1.185 | ± | 0.152 |
| Spleen | 1.692 | ± | 0.159 | 1.492 | ± | 0.144 | 6.561 | ± | 0.302 | 4.155 | ± | 0.345** |
| Small intestine (duodenum) | 0.535 | ± | 0.075 | 0.760 | ± | 0.156 | 2.085 | ± | 0.235 | 2.100 | ± | 0.397 |
| Kidney | 1.332 | ± | 0.068 | 1.555 | ± | 0.126 | 5.176 | ± | 0.047 | 4.338 | ± | 0.327 |
| **Fat** | 0.072 | **±** | 0.014 | 0.153 | **±** | 0.026 | **0.278** | **±** | **0.096** | **0.426** | **±** | **0.065** |
| Colon | 0.300 | ± | 0.023 | 0.296 | ± | 0.039 | 1.175 | ± | 0.017 | 0.826 | ± | 0.104 |
| Muscle | 0.103 | ± | 0.006 | 0.167 | ± | 0.006 | 0.401 | ± | 0.269 | 0.466 | ± | 0.014 |
| Bone marrow | 0.809 | ± | 0.079 | 0.781 | ± | 0.153 | 3.128 | ± | 0.234 | 2.146 | ± | 0.412 |
| Bone | 0.373 | ± | 0.061 | 0.561 | ± | 0.076 | 1.446 | ± | 0.095 | 1.561 | ± | 0.209 |
| Testes | 0.641 | ± | 0.031 | 0.673 | ± | 0.019 | 2.488 | ± | 0.181 | 1.886 | ± | 0.060 |
| Urine | 0.155 | ± | 0.048 | 1.719 | ± | 0.927*** | 0.598 | ± | 0.019 | 4.838 | ± | 2.561*** |
| Whole blood | 0.146 | ± | 0.004 | 0.160 | ± | 0.008 | 0.568 | ± | 0.028 | 0.448 | ± | 0.020 |
| **Plasma** | 0.182 | **±** | 0.007 | 0.201 | **±** | 0.011 | **0.708** | **±** | **0.018** | **0.560** | **±** | **0.027** |
| RBC | 0.088 | ± | 0.005 | 0.102 | ± | 0.005 | 0.343 | ± | 0.252 | 0.285 | ± | 0.011 |

**Brain time activity curves**

Post processing and kinetic modelling of the PET data was performed using PMOD software (v3.8; PMOD technologies, Zurich). CT images from individual rats were co-registered (rigid match) with an MR template (Schiffer T2 rat). The transformations were applied to individual PET scans and manually adjusted where required. 3D volumes of interest (VOIs) available on PMOD for the MR template (Px Rat W.Schiffer) were modified as below to obtain brain VOIs. Based on literature cortex (cingulate, frontal association, insular medial prefrontal, motor, orbitofrontal, somatosensory cortex regions from the PMOD template were included in this VOI) was of primary interest[18]. Based on autoradiography data, additionally ‘midbrain’ regions (midbrain, thalamus, hypothalamus, colliculi) and brainstem (pons & medulla) were chosen as potential high uptake regions. Further, cerebellar grey and white regions as potential reference regions and striatum and hippocampus as low uptake regions were also chosen for analysis. Gaps within the VOIs were closed, regions from the left and right hemisphere were merged and edges trimmed back from bone to obtain the final VOIs (Figure 1a and 1b). Bone VOIs were defined on the CT images of each rat by automatic, 3D, hot iso-contouring at 50% threshold. TACs were obtained for each of the VOIs and the results expressed as %ID/g. Additionally, the ratio between the region and cerebellar grey was calculated.

**Supplementary figure 1a.** Time-Activity Curves of various brain regions (left) and corresponding region to cerebellum ratios (right)





**Supplementary figure 1b.** Bone average TACs (without error bars for visual clarity) shown in the context of the brain regions in SHRwt (left) and SHR24 (right).





**Supplementary figure 1c.** Representative PET scan images (%ID/g, 50-60 min summed) of [^18^F]AV1451 uptake in the brains of SHRwt (left) and SHR24 (right) rats co-registered with an MRI template and ROIs visualised: cortex (blue), midbrain (pink), brainstem (red), striatum (orange), hippocampus (purple), cerebellum grey (yellow) and cerebellum white (green).

**
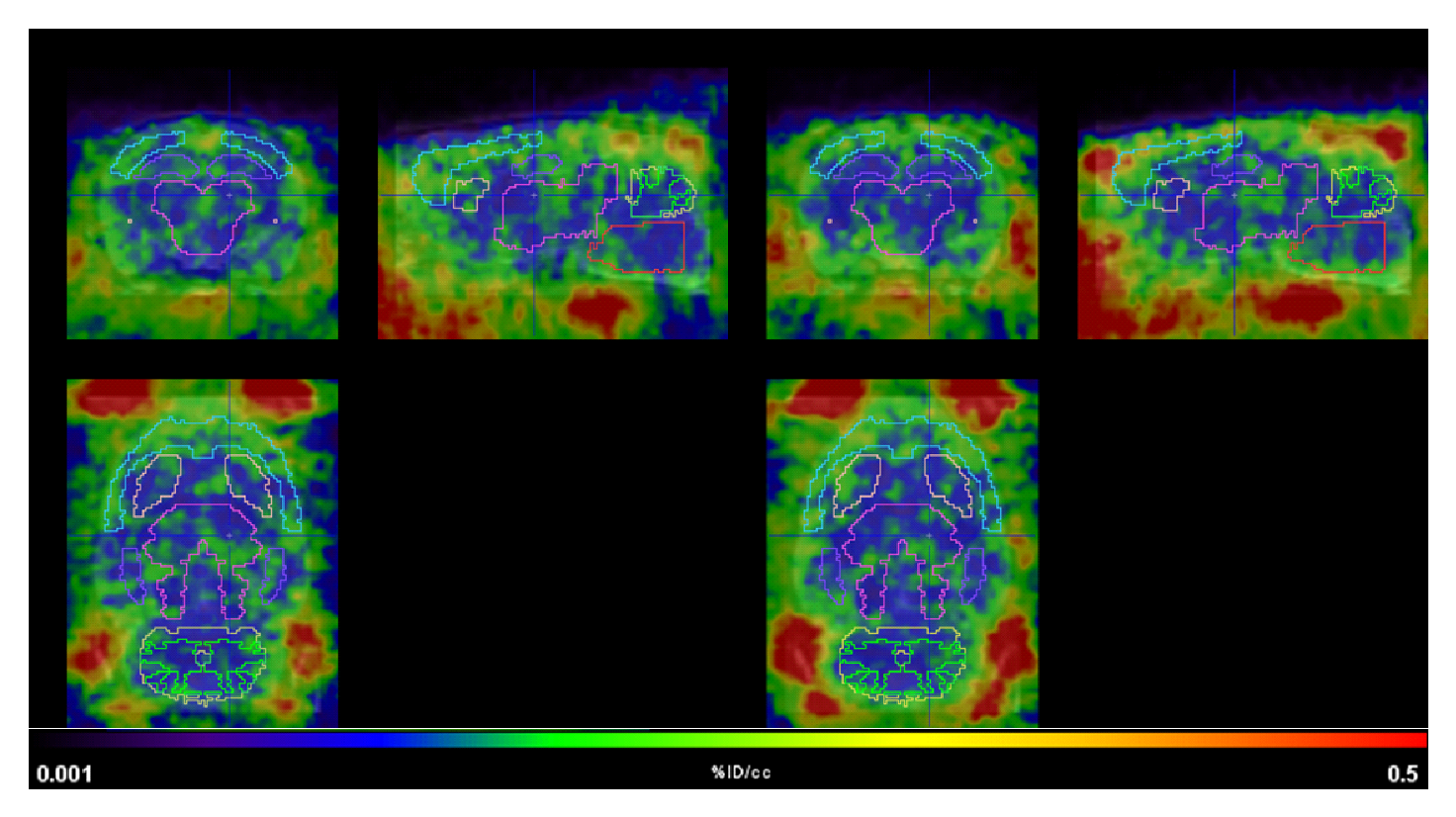
**

**Metabolite analysis**

An equal volume of ice-cold acetonitrile was added to the plasma and the mixture vortexed (5-10 seconds) and centrifuged (5 °C, 5 min, 30130 RCF). The supernatant was analysed by HPLC (Thermo Scientific Ultimate 3000 HPLC system with a Luna C18 5 μm 100 Å, 4.6 x 250 mm column held at 30 °C, fitted with a 200 μL loop). Solvent A was 20 mM NH4Oac pH 8.5 in H2O and Solvent B was acetonitrile. A linear gradient of 5 to 95% B in A at over 10 minutes, followed by holding at 95% B 2 minutes, followed by a re-equilibration at 5 % B for 3 minutes at a flow rate of 1.5 ml.min-1 was used (total run time 15 minutes). The eluate was passed through a Berthold Flowstar LB 513 radiodetector and 1.5 mL (1 minute) fractions were collected from the outlet and radioactivity present in the samples was measured with the gamma counter. The parent radioligand concentration was expressed as a percentage of the total plasma radioactivity.

**Supplementary figure 2.** (a) Metabolite curves, (b) its area under the curve, (c) metabolite corrected plasma curves, (d) its area under the curve, (e) representative example chromatograms obtained from the HPLC fractions from plasma samples at 5, 10, 15, 30 and 60 min of one rat.


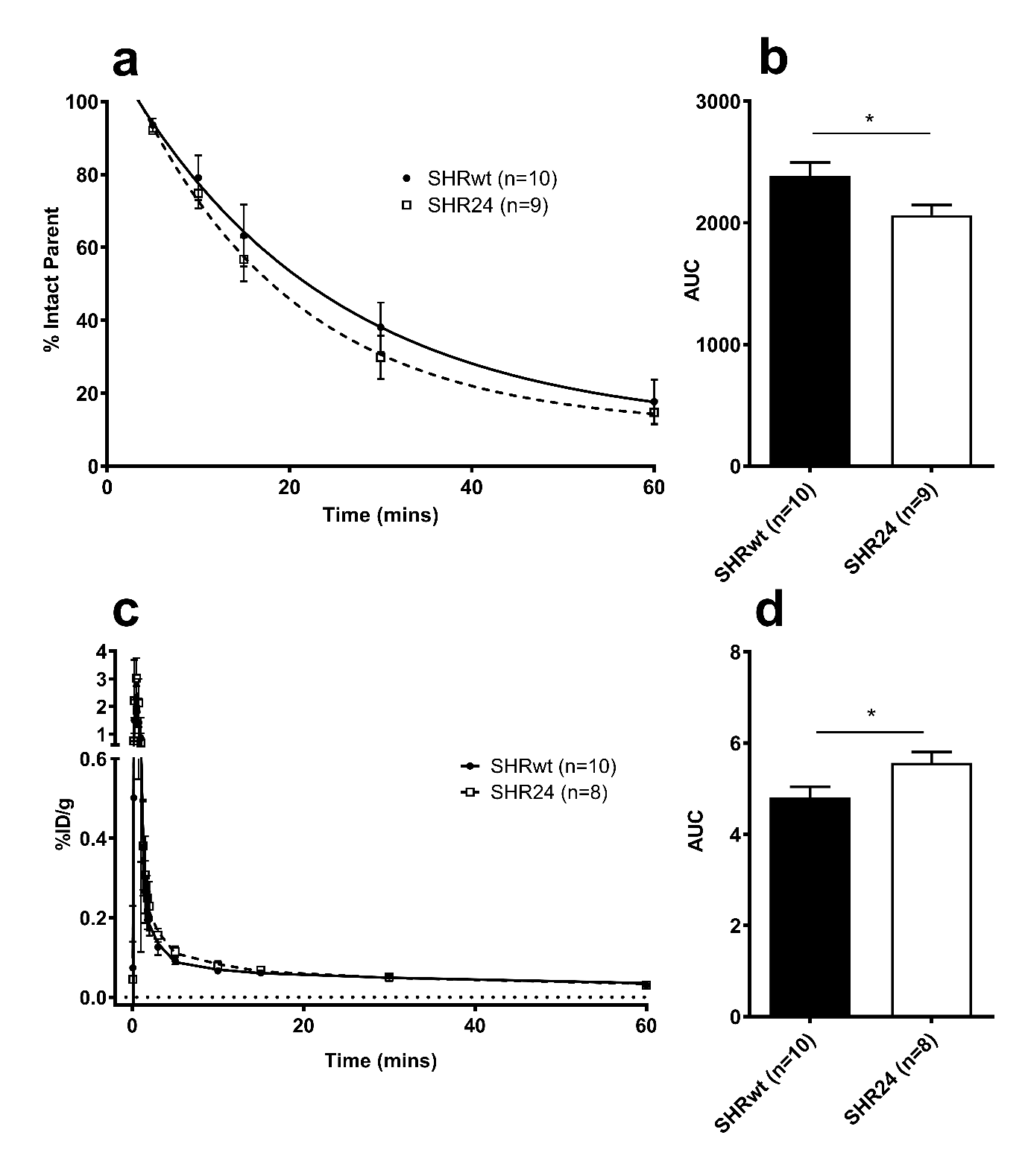





**Supplementary figure 3.** Comparison of k_3_/k_4_ values (a, c) before and (b, d) after values above one are defined as outliers. Significant differences between SHR24 and SHRwt animals became apparent in the case of cortex (b), but did not survive multiple comparison (d) when compared alongside other regions.








**Power Analysis**

The a priori power analysis was performed on data obtained from a pilot study (n=3) performed on an older scanner without CT. This analysis indicated that an n of 7 to 10 would be required for a 2-sample, two-tailed t-test at 80% power at an alpha of 5% for V_T_ and BP_ND_ of the whole brain and cortex regions.

A post-hoc power analysis performed for the 2-sample, two-tailed t-test for cortex after removing the values above one (graph b, shown above) returned an actual power of 0.8533 at an alpha of 0.05 using the below parameters.

| **Parameter** | **SHRwt** | **SHR24** |
| --- | --- | --- |
| Means | 0.3187 | 0.6354 |
| Sigma | 0.187 | 0.1736 |
| Sample size | 7 | 7 |

Lenth, R. V. (2006-9). Java Applets for Power and Sample Size [Computer software]. Retrieved August, 1st, 2024 from <http://www.stat.uiowa.edu/~rlenth/Power>.

With a Cohen’s d of 1.755, the effect size is large.

Effect size calculator for t-test. Retrieved August, 1st, 2024 from <https://www.socscistatistics.com/effectsize/default3.aspx>

**Supplementary figure 4.** Correlation between V_T_ from Logan graphical analysis and BP_ND_ from Simplified Reference Tissue Model.





**Fluorescence imaging**

The slices were fixed in 4% paraformaldehyde in PBS (20 min) and rehydrated with PBS (5 min). Slices were blocked with 5% BSA in PBS with 0.3% TritonX-100 and incubated overnight at 4 °C with AT8 monoclonal antibody, mouse IgG1 (Thermo Scientific, MN1020; 1:1000) in PBS with 0.3% TritonX-100. The slides were washed (3x 5 min) with PBS and incubated with Alexa Flour 647, Goat anti-mouse (Invitrogen, A-21235; 1:1000) in PBS with 0.3% TritonX-100 (2-3 hours) at room temperature. The slides were washed (3x 5 min) with PBS and further incubated with 10 μM of a fluorescent analogue of AV1451, T557[25] in PBS (1 hour) at room temperature and washed (3x 5 min) with PBS. Counterstaining was performed with DAPI dye (SigmaAldrich, MBD0015; 1:1000) (15 min) and washed (3x 5 min) with PBS. FluorSave reagent (Calbiochem) was applied to the slides, and slides were coverslipped. Slides were imaged with Leica Dmi8 inverted light microscope at 5X magnification for tile-scanned image of T557 binding and Leica DM6000 epifluorescent microscope at 40X magnification for visualisation of T557 and AT8.

**Supplementary figure 5- Fluorescence imaging.** The 5X tile scanned (**a**) T557 image revealed areas of higher fluorescence in the corresponding areas to those with high uptake seen in the autoradiography, namely cortex, midbrain and brainstem. The images at 40X revealed co-localisation of T557 uptake with that of AT8 within the same cells in (**b**) cortex, (**c**) midbrain and (**d**) brainstem. White arrows indicate examples of cells presenting both T557 and AT8 signal.


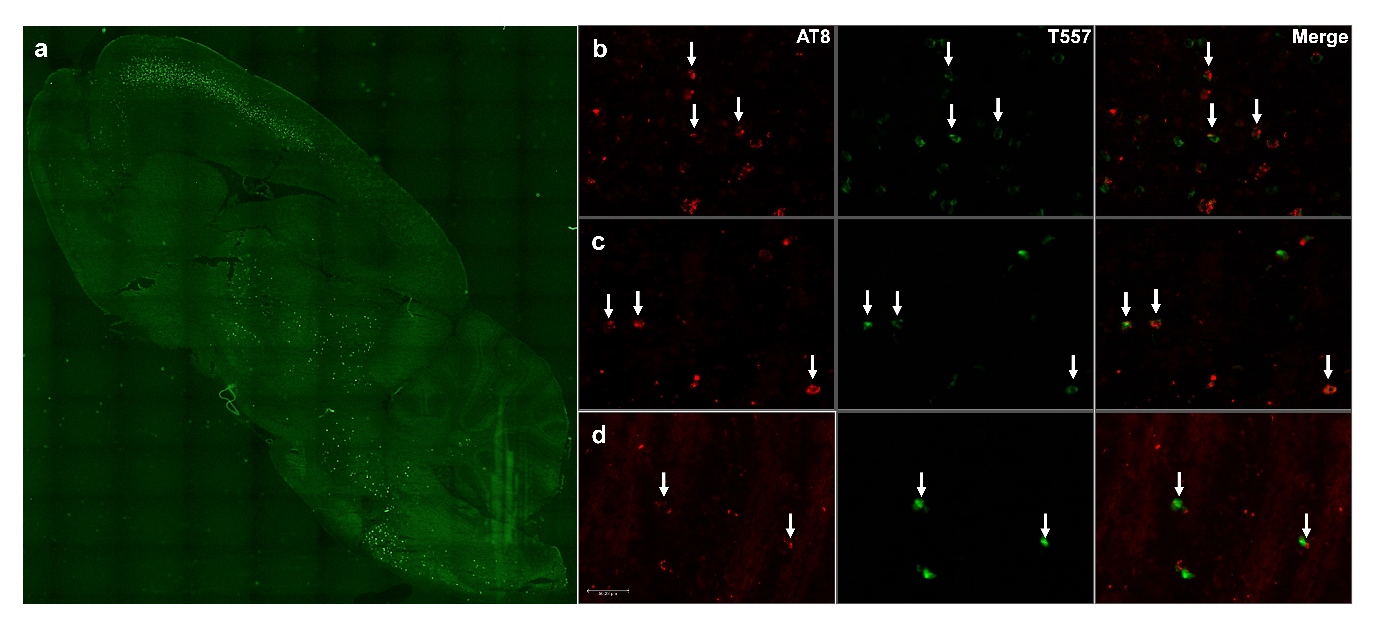


**Supplementary figure 6.** Autoradiography protocol (**a**) with and (**b**) without washes containing ethanol on brain slices from the same animal


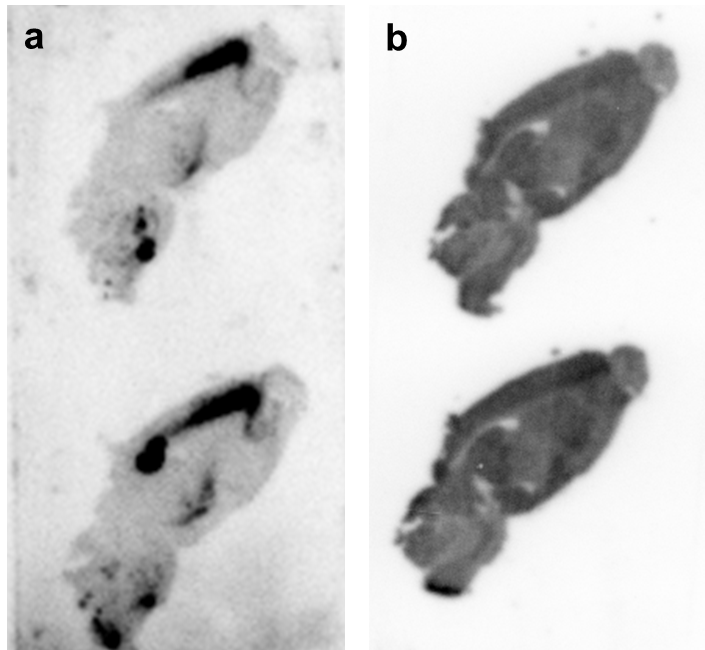

Supplement: Supplementary file 1 — Supplementary file1 (DOCX 13584 KB) [file 11307_2024_1972_MOESM1_ESM.docx]
